# Teledentistry—Dental students’ preparedness and patients’ experiences

**DOI:** 10.1371/journal.pone.0318991

**Published:** 2025-02-13

**Authors:** Swarna Math, Janice Diong Li Nga, Huey Fen Lim, Maryam Amin, Camila Pacheco-Pereira

**Affiliations:** 1 Mike Petryk School of Dentistry, University of Alberta, Edmonton, Alberta, Canada; 2 Ministry of Health Malaysia, Kampar District Dental Health Office, Kampar, Malaysia; 3 Ministry of Health Malaysia, Klang District Dental Health Office, Klang, Malaysia; Ajman University, UNITED ARAB EMIRATES

## Abstract

**Objectives:**

To explore dental students’ preparedness, experiences and confidence levels in utilizing teledentistry (TD) for patient care during the COVID-19 pandemic and to determine the dental needs and experiences of patients receiving treatment from dental students through TD.

**Methods:**

Two online surveys were administered through Google Forms, incorporating both five-point Likert scales and open-ended questions alone for the student questionnaire. The patient questionnaire, available in English and Mandarin, was distributed during or after teleconsultations. Descriptive statistics were used to summarise the data.

**Results:**

Out of 125 students, 88% (N = 110) were contacted by 318 patients. Of them, 79.1% reported no telecommunication problems, 91.8% faced no language barriers, and 87.2% encountered no technological barriers. Most students (67.3%) agreed they would need further training in TD. Low confidence levels were observed among 26.3% of students in managing crowns and bridges and among 18.2% of students in managing mucosal conditions. From the patient perspective, 76.4% contacted the students via WhatsApp Messages and 21.0% used the Voice Call mode. About 44.0% of patients enquired about the next available appointment. Their most common concern was tooth pain (15.1%) followed by denture problems (9.1%), chipped fillings (6.6%), and crowns and bridge problems (6.3%). Overall, 82% patients reported effective communication during teleconsultation, 85% were satisfied with the questioning process, and expressed satisfaction with the diagnoses provided. However, 10% of patients chose to ignore their concerns, and 5% sought assistance from medical practitioners.

**Conclusion:**

Most dental students were confident in addressing patient concerns but recognized the need for additional training for managing complex cases. Both students and patients reported positive experiences with TD, including effective communication and satisfaction. These findings highlight the importance of integrating TD training into dental curricula, addressing technical and privacy concerns, and improving patient education for secure and effective TD use in routine care.

## Introduction

Teledentistry (TD) innovatively challenges the traditional in-person doctor-patient model by enabling remote diagnosis and treatment through video conferencing. It offers an effective alternative to address challenges such as limited access during health emergencies like the COVID-19 pandemic and resource constraints on in-person appointments [1,2]. The American Dental Association refers Telehealth as to “the broad variety of technologies and tactics to deliver virtual medical health, and education services. TD is not a specific service, but a collection of means to enhance care and educational delivery” [3]. A virtual appointment can take place anywhere as there is a video teleconference capability. The travel to a distant clinician or a specialist is decreased or eliminated, and the referral-to-visit may be shortened [4]. Implementing such programs particularly during the pandemic can cost-effectively ensure patient and clinician safety, while also reducing strain on healthcare facilities [5].

It is widely recognized that the pandemic severely disrupted the provision of health services, including dental services, because of the prioritization of treating COVID-19-positive patients and controlling the transmission of infections [6,7]. During this global pandemic, TD emerged as a useful tool for bridging the gap between dentists and patients [8]. This enabled patients to stay at home and connect with physicians through information and communication technology, reducing the spread of the virus among the community and the frontliners [1,9]. Many people were likely to forego non-emergency dental procedures due to the limitations placed on the provision of dental care as well as the added financial and psychological strain brought on by the COVID-19 pandemic [10]. TD holds considerable value from the patient perspective [11]. Few studies have demonstrated that patients appreciated the accessibility of teleconsultations during restricted access periods, with many findings it is effective for addressing their oral health concerns and providing interim solutions [6,8,11].

Globally, TD has yet to be fully integrated in dental education [12]. A Canadian study found that only 30% of dental programs and 62.5% of dental hygiene programs included TD, mainly through lectures with minimal practical application [12]. These studies highlight a pressing need to better prepare students for modern dental practice, as challenges like overcrowded curricula, limited faculty expertise, and technological barriers continue to impede its adoption [13]. To the best of our knowledge, there is limited evidence regarding dental students’ preparedness and experience with TD. Additionally, telehealth remains underexplored from patient perspectives, necessitating further studies to evaluate their experiences and satisfaction. The objectives of this study were to assess the preparedness, experiences and confidence levels of dental students in utilizing TD for patient care, and to determine the dental needs and experiences of patients receiving treatment from dental students through TD.

## Materials and methods

### Study design

This cross-sectional study was approved by the University Joint Committee on Research and Ethics, International Medical University, Kuala Lumpur, Malaysia (IMU-JC 501/2020) and conducted in accordance with the Declaration of Helsinki. The institution offers a five-year DDS curriculum, with clinical training beginning in the third year. The study population included Year 3 (n = 50), Year 4 (n = 46), and Year 5 (n = 46) dental students who had resumed clinical activities on campus following COVID-19 restrictions. Additionally, patients who consulted with these students during teleconsultations were included in this study. While the study was limited to a single institution, the findings provide valuable insights but may not generalize to the broader undergraduate dental or patient populations in Malaysia. The recruitment period for this study spanned from 23/11/2020 to 30/03/2021.

Two online surveys were conducted assessing dental students and a second one for patients involved in teleconsultations. Both surveys were conducted using Google Forms, and written informed consent was obtained from all participants through the form before they proceeded to complete the questionnaire. To ensure confidentiality, all responses were anonymized, and participation was voluntary. During teleconsultations, students used secure platforms like WhatsApp with safeguards, avoided sharing identifiable information, obtained verbal consent, and excluded identifying details from shared images. They were instructed to delete patient-related communications post-consultation, following institutional privacy policies. For the video calls, a reserved space was prepared for this purpose, warranting patient confidentiality.

### Survey instrument

The questionnaires were validated by Pelaso RM et al. [6] and Rahman N et al. [8] and adapted as per the study inclusion criteria. The modification of the questionnaire involved a face-to-face meeting with a group of five Year 5 students to qualitatively explore the scope of patients contacting students during the period of social distancing. The revised questionnaires underwent an institutional review to ensure internal validity and alignment with study objectives, though external validation was not conducted.

The student questionnaire included a total of 12 questions with details on socio-demographics, the number of patients who contacted the students during the periods of movement restriction periods, modes of telecommunication, types of oral health problems/concerns reported by the patients, language barriers, and technical issues experienced by students when responding to patients. Additionally, the survey assessed students’ confidence levels and challenges in telediagnosis and tele-management of patients’ concerns. Responses were measured using a five-point Likert scale, with options ranging from 1 (strongly disagree) to 5 (strongly agree). Open-ended questions captured students’ self-perceived challenges in TD. The complete version of the questionnaire is presented in S1 Appendix.

The patient questionnaire comprised 11 questions addressing patient demographics, oral health concerns, ease of use of TD, effectiveness in addressing concerns, satisfaction levels, and actions taken post-consultation. Students facilitated the completion of the survey when needed. The complete version of the questionnaire is presented in S2 Appendix .

### Survey distribution

The survey was emailed to the students with a link to an online survey tool (Google Form**©)** via institutional student emails. They were requested to submit the questionnaire in one week. As a strategy to increase the response rate, two reminders were sent after one week and at the end of the second week via emails and personal contacts.

For the patient questionnaire, students distributed the survey to their patients during or after teleconsultations with a link to an online survey tool (Google Form©). The questionnaire was available in both English and Mandarin to accommodate the predominantly Chinese patient flow and ensure inclusivity. Patients were provided verbal instructions to complete the survey, and assistance was offered when necessary. Verbal consent was obtained prior to participation. Responses were anonymized, and all identifiable information was excluded to maintain confidentiality.

### Statistical analysis

Data from the student and patient questionnaires were analyzed using IBM SPSS (version 26.0). We tabulated the number and percentage of students who were contacted by their patients, and the total number and mean number of patients who were contacted. The methods of communication used were reported. The frequency distributions for the reasons for contacting were calculated. We reported on the experience of the students when responding to the patients and calculated the frequency distributions for their confidence levels. The students’ need for training in telecommunications was described. Similarly, patient responses were analyzed to assess the ease of use, effectiveness, and satisfaction with TD, as well as actions taken post-consultation.

## Results

A total of 125 dental students participated in the survey, with an 88% response rate, of whom, 38 (30.4%) were in Year 3, 41 (32.8%) in Year 4, and 46 (36.8%) in Year 5. Thirty (24%) were male and 95 (76%) females. A total of 110 (88%) students reported that they were contacted by 318 patients, with an average of 3 patients per student.

### Modes of telecommunication used in teledentistry

**Fig 1:**
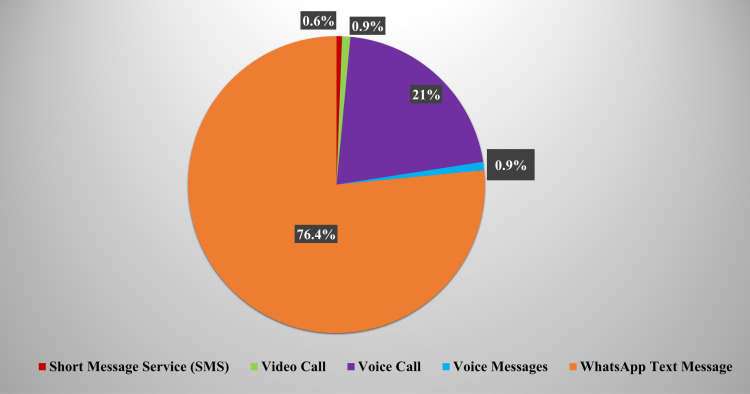
Mode of Communication Used in Teledentistry.

Most patients, 243 (76.4%), communicated using WhatsApp messaging, whereas 67 (21%) used Voice Call Mode, 3 (0.9%) used Voice Messages and Video Calls, and only 2 (0.6%) Short Message Service was the least popular, with only 2 (0.6%) patients choosing to use it as a mode of telecommunication (Fig 1).

### Oral health problems/concerns reported by patients

Of 318 patients who contacted, 140 (44.0%) enquired about the next available appointment. (Table 1) The other common problems reported were tooth pain (48, 15.1%), denture problems (29, 9.1%), chipped fillings (21, 6.6%), and crowns/bridges problems (20, 6.3%).

**Table 1:** Oral health concerns reported by patients during teleconsultation.

Oral Health Concerns	N (%)
Enquiry on the next available appointment	140 (44.0)
Tooth pain	48 (15.1)
Denture problem	29 (9.1)
Chipped off filling	21 (6.6)
Crown/ Bridge problem	20 (6.3)
Hole in the tooth	20 (6.3)
Mobile tooth/ Removal of tooth	12 (3.8)
Gum swelling	10 (3.1)
Bleeding gums	6 (1.9)
Ulcer in the mouth	2 (0.6)
Others	10 (3.2)

### Students’ experience of teledentistry

**Fig 2:**
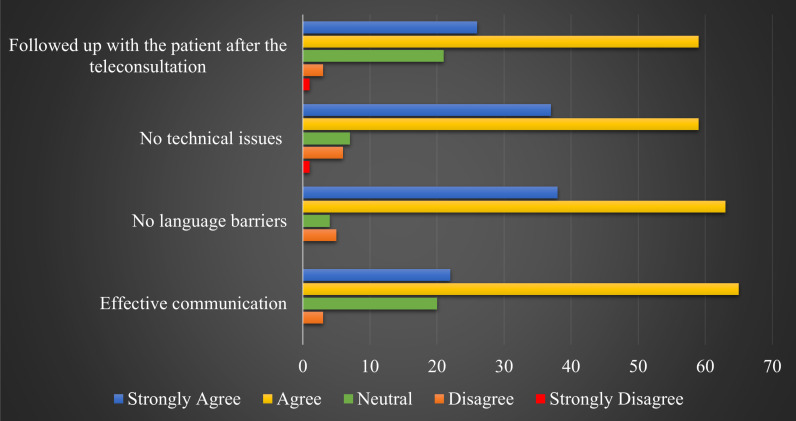
Students’ experience of teledentistry.

The feedback on the experience of TD was very positive. A majority of 87 (79.1%) students mentioned that they could communicate effectively with their patients during teleconsultation. Of the 110 students, 101 (91.8%) agreed or strongly agreed that there were no language barriers. Ninety-six (87.2%) reported that there were no technical issues encountered such as phone issues and internet access issues and 85 (77.0%) followed up with their patients’ concerns after the first teleconsultation (Fig 2).

Responses to open-ended questions suggested numerous challenges faced by students during TD. These included “addressing patient’s anxiety regarding their treatment’‘, “lack of response by patients” and “uncertainties about treatment outcomes due to delay in appointment.” Other comments were “I found difficulty in diagnosing the patient’s oral condition as the explanation or pictures provided by the patients were not clear”.

### Confidence level in telediagnosis and telemanagement

Seventy-seven (70.0%) agreed or strongly agreed that they were confident in the way they responded to their patients, with 61 (55.0%) reporting confidence in telediagnosis. However, 74 (67.3%) agreed on the need for TD training, see Table 2.

**Table 2:** Confidence level of dental students in telediagnosis.

Statement	N (%)				
	SD	D	N	A	SA
I was confident in the way I responded to the patient’s concern	0 (0)	6 (5.5)	27 (24.5)	64 (58.2)	13 (11.8)
I was confident with the diagnosis and explanation that I provided for the patient	2 (1.8)	9 (8.2)	38 (34.5)	51 (46.4)	10 (9.1)
I was confident in the way I managed the patient’s concern	0 (0)	11 (10.1)	25 (22.7)	60 (54.5)	14 (12.7)
I would require training for teledentistry in the future	1 (0.9)	2 (1.8)	33 (30.0)	51 (46.4)	23 (20.9)

SD =  Strongly Disagree, D =  Disagree, N =  Neutral, A =  Agree, SA =  Strongly Agree.

The students expressed their confidence level in the telemanagement of various oral health conditions. As per Table 3, higher confidence in tele management was expressed for tooth pain 68 (61.9%), gum swellings −58 (50.6%) and bleeding gums −81 (73.6%) than for denture problems −39 (35.4%), crowns and bridges problems −29 (26.3%) and burning mouth sensation −20 (18.2%).

**Table 3:** Confidence level of dental students in telemanagement.

Oral Conditions	N (%)				
	SNC	NC	N	C	SC
Tooth Pain	2 (1.8)	11 (10.0)	29 (26.4)	61 (55.5)	7 (6.4)
Gum Swelling	1 (0.9)	14 (12.7)	37 (33.6)	51 (46.4)	7 (6.4)
Bleeding Gums	0 (0)	5 (4.5)	24 (21.8)	70 (63.6)	11 (10.0)
Denture Problems	2 (1.8)	20 (18.2)	49 (44.5)	34 (30.9)	5 (4.5)
Crown/Bridge Problems	3 (2.7)	24 (21.8)	54 (49.1)	27 (24.5)	2 (1.8)
Chipped off filling	0 (0)	7 (6.4)	27 (24.5)	64 (58.2)	12 (10.9)
Hole in tooth	1 (0.9)	4 (3.6)	32 (29.1)	60 (54.5)	13 (11.8)
Mobile Tooth/Tooth Removal	0 (0)	9 (8.2)	29 (26.4)	62 (56.4)	10 (9.1)
Mouth Ulcer	1 (0.9)	16 (14.5)	44 (40.0)	44 (40.0)	5 (4.5)
Burning Mouth Sensation	6 (5.5)	23 (20.9)	61 (55.5)	18 (16.4)	2 (1.8)

SNC =  Strongly not confident, NC =  Not confident, N =  Neutral, C =  Confident, SC =  Strongly Confident.

### Patients’ experience with teledentistry

Responses from the 318 patients highlighted their experiences with teleconsultations during COVID-19 lockdowns, addressing various oral health concerns and providing insights into communication effectiveness, ease of use, and satisfaction levels. The patient responses reveal a generally positive experience with TD. A majority of patients (N = 261;82%) indicated that teleconsultation enabled effective communication with dental students, with interactions deemed comparable to in-person consultations. Moreover, 90% (N = 286) of patients expressed comfort in articulating their concerns in their preferred language during the teleconsultation process. Technical challenges were minimal, reported by 24% (N = 76) of the patients, and 68% (N = 216) of the patients confirmed successful follow-ups. Satisfaction levels were high, with 85% (N = 270) satisfied with students’ questioning to understand their concerns and 78% (N = 248) pleased with the explanations and diagnoses provided. Similarly, 74% (N = 235) were satisfied with the management of their oral health concerns, and 81% (N = 258) indicated a willingness to use teleconsultation in the future. Following teleconsultation, it was noted that 42% (N = 134) sought further management at dental clinics, 25% (N = 80) reported relying on over-the-counter medications, and 18% reported engaging in self-medication. However, 10% (N = 32) of the patients opted to ignore their concerns, and 5% (N = 16) of them sought assistance from general medical practitioners. Despite the overall positive experience, 24% (N = 76) of the patients highlighted certain challenges, including difficulties in obtaining clear intraoral images and uncertainties arising from delays in treatment appointments during restricted access to care.

## Discussion

Teledentistry (TD) is relatively incorporated in oral healthcare services, yet telehealth has long been utilized in medicine and has shown limited use in dentistry. Due to the growth of technological capabilities, TD has the capability to change the current dental practice [14]. This study focused on assessing the preparedness, experience, and confidence of dental students in utilizing TD for patient care while capturing patient experiences and satisfaction with the process during the COVID-19 lockdown. The findings reveal important insights into the effectiveness of TD, with most students demonstrating preparedness but expressing a need for further training to enhance their confidence and proficiency in managing diverse oral health concerns remotely. Simultaneously, patients reported largely positive experiences, emphasizing effective communication and satisfaction with the diagnostic and management aspects of TD. These results address critical gaps in understanding the role of TD in dental education and practice, proposing solutions such as structured training programs that combine theoretical and practical components. By fostering proficiency in remote care, TD offers innovative solutions to ensure continuity of care in emergencies and resource-limited settings. This study contributes to the growing body of evidence supporting TD’s integration into dental curricula and clinical practice, paving the way for its broader implementation and potential to bridge healthcare disparities.

Teledentistry is subdivided into teleconsultation, telediagnosis, and telemonitoring. Teleconsultation may facilitate patient care during quarantine and lockdown [15]. Teleconsultation can be done through instant messaging or video calling [5]. In our study, most patients opted for WhatsApp Messages as the primary mode of communication, reflecting its accessibility and global preference in telehealth for its ease of use and familiarity [5] while seldom chose video calls for teleconsultation. While privacy concerns have been reported as a significant barrier to TD adoption and its effective implementation [16,17], the use of secure platforms here minimized such risks, highlighting the importance of institutional policies for safeguarding confidentiality. However, reliance on general messaging platforms may limit diagnostic precision compared to specialized telehealth tools, suggesting the need to integrate dedicated platforms into dental education to enhance diagnostic accuracy and care efficiency [12].

During teleconsultation, most patients’ inquiries focused on scheduling appointments, reflecting anxiety over completing ongoing dental treatments. In this study, fewer concerns (15.1%) were related to emergency issues such as odontogenic pain, emphasizing the role of telehealth in distinguishing emergency from non-emergency care. These findings align with studies highlighting teleconsultation’s effectiveness in addressing patient concerns, optimizing triaging, and maintaining continuity of care during disruptions [12,16]. Such situations reinforce TD’s potential to alleviate patient anxiety and ensure timely care during limited access to in-person consultations.

In the present study, dental undergraduates’ experiences in the field of TD were generally positive, with minimal technical issues reported (12.8%). The smoother experience observed in this study may be attributed to the availability of advanced digital infrastructure in urban settings, as patients were primarily city residents. In contrast, previous studies have reported challenges with internet-related equipment, as well as significant barriers such as cost and lack of technological knowledge, hindering the broader adoption of TD [5,18,19]. Although most students demonstrated higher confidence in addressing routine dental concerns, such as “tooth pain” and “gum swelling”, their confidence was lower when managing more complex cases, such as any problems with crowns and bridges. This disparity highlights the lack of formalized training in TD and limited exposure to advanced case management during undergraduate education, which is consistent with previous authors observations [12,13]. Structured TD modules that incorporate simulation-based training and interdisciplinary approaches are essential for enhancing student preparedness and confidence in diagnosing and managing diverse oral health conditions [12,13,20].

Teleconsultation reduces non-emergency patient referrals, appointments and eases the burden on healthcare frontliners with patient load [15]. In this study, nearly half of the students provided reassurance to their patients during teleconsultations, no clinical follow-up were reported for 12% of the cases. Acute pain cases were effectively managed by prescribing over-the-counter medications or referring patients to dental practitioners, a practice consistent with findings from prior study highlighting TD’s role in streamlining referrals during crises [20]. Teleconsultation’s effectiveness in triaging patients and streamlining referrals has been supported by a review highlighting its role in reducing in-person visits and maintaining care continuity [16]. As Hung et al. [16] and Peanchitlertkajorn et al. [17] recommended robust institutional policies and secure platforms are crucial for addressing privacy concerns and building patient trust, while targeted training in teleconsultation and diagnostics can further enhance TD’s efficiency in modern dental practice. Interestingly, none of the patients in this study expressed concerns about privacy during teleconsultation, which may reflect trust in the process or limited awareness of privacy risks. In contrast, Peanchitlertkajorn et al. [17] identified confidentiality and privacy as significant concerns, with risks such as unauthorized recording and sharing of consultations. Anecdotal evidence suggests students may also undervalue privacy, increasing the risk of breaches. These findings highlight the importance of integrating data privacy training into TD curricula and call for future research to assess privacy awareness among patients and providers to enhance confidentiality in telehealth. Telemonitoring, an emerging aspect of TD, reduces the necessity for routine face-to-face dental visits by enabling virtual follow-ups of oral health conditions [5]. In the present study, most of the students followed up with their patients’ concerns after the initial teleconsultation, highlighting telemonitoring’s practicality in ensuring care continuity during extended lockdowns and social distancing mandates. Similar observations in the literature have emphasized the potential of telemonitoring to enhance chronic disease management and reduce healthcare burdens, particularly in underserved regions [16,21]

This study provides valuable insights into patient experiences with TD during pandemic times, demonstrating its effectiveness in maintaining communication and addressing basic oral care needs. While technical challenges, such as difficulties with intraoral imaging, were minimally reported (24%), they highlight inherent limitations in remote diagnosis that have been highlighted across studies [8,11], emphasizing the need for better imaging tools and patient-clinician training.

The predominance of non-emergency concerns, such as appointment inquiries, supports TD’s role as a triage tool rather than a replacement for in-person care. This aligns with global trends indicating patient reliance on TD for initial assessments while maintaining a preference for face-to-face interactions, as emphasized in previous studies [6,11,22]. Additionally, the finding that 10% of patients ignored their concerns suggests the need for enhanced follow-up and referral mechanisms. The mix of follow-up actions, including 42% seeking clinic visits and 43% relying on self-management, reflects TD’s dual role as a triage and advisory tool. These findings highlight its potential to complement traditional care while emphasizing the importance of addressing limitations for broader integration into routine dental services. The results of our study bolster the advantages of incorporating TD into both dental education and dental practice. It even raised the question, “Are we preparing upcoming dentists to handle TD effectively?” Hence, given the expanding scope of technology and the increasing technological literacy across generations, it is imperative for dental schools to proactively integrate TD into their curriculums. Students’ confidence and preparedness must be integrated, particularly in light of any health emergencies. Furthermore, the positive patient experiences, including high satisfaction with communication and care, emphasize the potential of TD to improve access to dental services. Addressing patient-reported challenges, such as technical barriers and the need for clearer follow-up guidance, will be vital in ensuring TD’s broader acceptance and long-term sustainability.

### Strengths and limitations

With a high response rate of 88%, this study evaluates dental students’ preparedness, confidence and experiences in TD during the pandemic, offering insights into their challenges and training needs. Its results emphasised TD’s role in maintaining care continuity and addressing patient concerns. The inclusion of patient experiences provides a comprehensive understanding of TD’s effectiveness in communication, patient satisfaction, and its role as a triage tool during restricted access to care.

As limitations, the lack of external validation and potential response biases may affect the reliability of the findings. To mitigate these biases, validated questionnaires were utilized [6,8]. The lack of formal assessment of privacy concerns among patients and students during teleconsultations highlights the need for further research into privacy awareness and potential breaches in dental teleconsultation settings. Also, these results cannot be extrapolated to all DDS students, as the study was conducted within a single institution, reflecting the experiences of a specific cohort in an urban setting. The findings may not capture the diversity of dental education systems, curricula, or the unique challenges faced by students and patients in different countries and regions.

### Future directions

Scholarly, a comprehensive framework can be proposed to effectively integrate TD into dental education, combining theoretical and practical training components to equip students with essential skills for remote care. Foundational knowledge can be delivered through lectures on telehealth principles, ethics, legal frameworks, and data privacy.

On a practical skills approach, it can be developed through supervised clinical rotations, allowing students to apply theoretical concepts in real-world contexts. Pilot training programs can be implemented to assess the feasibility and effectiveness of integrating TD into existing curricula. Collaboration with public health and IT professionals is also recommended to prepare students for managing complex remote care scenarios. Incorporating these strategies into dental curricula could help address health care inequalities in underserved areas, enhance care efficiency, and improve patient outcomes.

## Conclusion

The findings reveal that most dental students were prepared to utilize TD, with 70% expressing confidence in addressing patient concerns and 55% confident in providing accurate diagnoses. However, students identified the need for further training to enhance their proficiency, particularly in telemanagement of complex oral health conditions. Patient experiences were predominantly positive, with 82% reporting effective communication during teleconsultations and 85% expressing satisfaction with how their concerns were addressed. Furthermore, 81% indicated a willingness to use TD services in the future, reflecting its potential to improve patient engagement and non-emergency oral healthcare delivery. These findings highlight the importance of integrating structured TD training into undergraduate dental curricula, combining theoretical knowledge with practical applications to prepare future dentists for effective remote care delivery. Addressing challenges such as licensure, legal considerations, and privacy concerns is critical to ensuring the successful implementation of TD in dental education and practice.

## Supporting information

S1 Appendix Student Questionnaire. (PDF)

S2 Appendix Patient Questionnaire. (PDF)
