# Supplementary material for: Teledentistry—Dental students’ preparedness and patients’ experiences
Source: PLoS One. 2025 Feb 13;20(2):e0318991. doi: 10.1371/journal.pone.0318991 (PMC11824952; doi:10.1371/journal.pone.0318991)
Supplement: S1 Appendix — (PDF) [file pone.0318991.s001.pdf]

Appendix 1: Student Questionnaire

1. Student ID (last 6 digits) ___________________

2. Gender: ☐ Male ☐ Female

3. Year of study: ☐ Year 3 ☐ Year 4 ☐ Year 5

4. Did any of your patients contact you during the lockdown period?

    ☐ Yes *(Proceed to Question 5)*

    ☐ No *(Proceed to Question 12)*

5. If yes, how many patients contacted you during the COVID-19 lockdown ____

6. What was the mode of communication with your patient? *More than one option can be selected.

    ☐ Voice call

    ☐ Short message service (SMS)

    ☐ WhatsApp text message

    ☐ Web audio/ video call

7. What were your patient's concerns? *More than one option can be selected.

    ☐ Enquiry on the next available appointment

    ☐ Tooth pain

    ☐ Gum swelling

    ☐ Bleeding gums

    ☐ Denture problem

    ☐ Crown/bridge problem

    ☐ Chipped off filling

    ☐ Hole in the tooth

    ☐ Mobile tooth / Removal of tooth

☐ Ulcer in the mouth

☐ Burning mouth sensation

☐ Others, please specify ______________

Please select your response (✓) regarding the statements given below.

	Strongly agree	Agree	Neutral	Disagree	Strongly Disagree	Not applicable
i. I was able to communicate effectively with the patient during teleconsultation.						
ii. There were no language barriers during the teleconsultation.						
iii. There were no technical issues during the teleconsultation. (e.g phone reception issues, internet access issues, picture taken by the patient is not clear)						
iv. I followed up with the patient after the teleconsultation.						

9. Please specify if you encountered any other challenges during the teleconsultation,

____

10. Please select your response (✓) about the statements below.

	Strongly agree	Agree	Neutral	Disagree	Strongly Disagree	Not applicable
i. I was confident in the way I responded to the patient's concern.						
ii.I was confident with the diagnosis and explanation I provided for the patient.						
iii.I was confident in the way I managed the patient’s concerns.						
iv.I am confident about teledentistry in the future.						
v.I would require training for teledentistry in the future.						

11. How did you manage your patient’s concerns?

☐ Refer to a general medical practitioner

☐ Refer to general dental practitioner

☐ Refer to IMU Oral Health Care

☐ Prescribe over-the-counter drugs

☐ Provide reassurance

12. Please select your response (✓) regarding your confidence in managing your patient's concerns are given below.

	Strongly Confident	Confident	Neutral	Not Confident	Strongly Not Confident
i. Tooth pain					
ii. Gum swelling					
iii. Bleeding gums					
iv. Denture problems					
v. Crown/bridge problems					
vi. Chipped off filling					
vii. Hole in tooth					
viii. Mobile tooth/ tooth removal.					
ix. Mouth ulcer					
x. Burning mouth sensation					
