# Supplementary material for: Teledentistry—Dental students’ preparedness and patients’ experiences
Source: PLoS One. 2025 Feb 13;20(2):e0318991. doi: 10.1371/journal.pone.0318991 (PMC11824952; doi:10.1371/journal.pone.0318991)
Supplement: S2 Appendix — (PDF) [file pone.0318991.s002.pdf]

Appendix 2: Patient Questionnaire

1. Year of birth 出生年份

   ______

2. Gender （性别）
   a. Male （男性）
   b. Female （女性）

3. Nationality （国籍）
   a. Malaysian （大马公民）
   b. Non-Malaysian （非大马公民）

4. Education level (教育程度)
   a. Primary (小学)
   b. Secondary (中学)
   c. Tertiary (大学)

5. Did you contact any IMU dental students during the COVID-19? (您是否曾在行动管制令期间联络过IMU的牙科学生？）
   a. Yes（有）
   b. No （没有）

6. Did you undergo any dental treatment in IMU prior to the lockdown? （在行动管制令开始前3个月， 您是否在IMU的牙科诊所接受任何牙科治疗？）
   a. Yes （有）
   b. No（没有）

7. What mode of communication did you use to contact the dental student during the lockdown? （您使用哪个沟通模式来联络学生？）
   a. Voice call （电话联络）
   b. Short message service (SMS) （手机短信）
   c. Whatsapp text message （Whatsapp 短信）
   d. Web audio/ video call （视频通话）

8. What was the reason for contacting the student during the lockdown?（您联络学生的原因是什么？）
   a. I wished to arrange a dental appointment.（我想预约）

b. I had tooth pain.（ 我的牙齿痛）
c. I had swollen gums.（我的牙龈肿）
d. I had bleeding gum. （我的牙龈流血)
e. I had denture problem.（我的假牙有问题）
f. I had a crown/bridge problem.（我的牙冠/牙桥断裂）
g. My filling chipped off.（我的牙齿崩了）
h. I had a hole in my tooth.（我的牙齿有洞）
i. My tooth was shaky/ I want to pull out my tooth. （我的牙齿松/ 我想拔牙）
j. I had an ulcer in my mouth.（我的口腔溃疡）
k. I had burning sensation in my mouth（我的嘴里有灼烧感）
l. Others, please specify （如果有其他，请注明） _______________

9. Please select your response (✓) in regards to the statements given below.

	Strongly agree	Agree	Neutral	Disagree	Strongly Disagree
I was able to communicate effectively with the student as well as if meeting in person.					
I was able to express myself effectively in the language I feel comfortable with during the teleconsultation.					
I was able to overcome any technical issue during the teleconsultation. (e.g phone reception issues, internet access issues, pictures taken as instructed by the student).					
I was able to follow up with the student after					

teleconsultation.					

10. Please select your response in regards to the statements given below.

	Strongly agree	Agree	Neutral	Disagree	Strongly Disagree
I am satisfied with how the student asked me questions to understand my concerns.					
I am satisfied with the diagnosis and explanation provided by students.					
I am satisfied with how the students manage my problem.					
I am willing to have teleconsultation in the future.					

11. How did you manage your condition after the consultation? （您在线上沟通后如何处理您的问题？）
    a. Ignore the problem 忽视那个问题
    b. Self-medicament 自行用药
    c. Over-the-counter medication 到药房拿药
    d. Sought help from general medical doctor 向医生求助
    e. Attended to the dental clinic for further management 到牙科诊所接受治疗
